# N^6^‐methyladenosine (m^6^A) RNA modification of G protein‐coupled receptor 133 increases proliferation of lung adenocarcinoma

**DOI:** 10.1002/2211-5463.13244

**Published:** 2022-01-26

**Authors:** Guixiong Wu, Dongfeng Zhai, Jiemei Xie, Shuiquan Zhu, Zhuo Liang, Xin Liu, Ziwen Zhao

**Affiliations:** ^1^ Department of Respiratory Medicine The First Affiliated Hospital of Jinan University Guangzhou China; ^2^ Respiratory Department The People’s Hospital of Wuzhou China; ^3^ Affiliated Cancer Hospital & Institute of Guangzhou Medical University China; ^4^ Department of Clinical Laboratory Guangzhou Chest Hospital China; ^5^ Department of Pulmonary and Critical Care Medicine Guangzhou First People’s Hospital The Second Affiliated Hospital of South China University of Technology Guangzhou China

**Keywords:** cell cycle, G protein‐coupled receptor 133, lung adenocarcinoma, proliferation

## Abstract

Lung adenocarcinoma (LUAD) accounts for almost 40% of lung cancers, leading to significant associated morbidity and mortality rates. However, the mechanism of LUAD tumorigenesis remains far from clear. Here, we scanned down‐regulated genes involved in LUAD sourced from The Cancer Genome Atlas and Gene Expression Omnibus data and focused on G protein‐coupled receptor 133 (*GPR133*). We offer compelling evidence that *GPR133* was expressed at low levels in the setting of LUAD, and higher expression was positively related to a better prognosis among patients with LUAD. Functionally, *GPR133* inhibited cell proliferation and tumor growth *in vitro* and *in vivo*. Regarding the mechanism, flow cytometry assays and western blot assays showed that *GPR133* enhanced p21 and decreased cyclin B1 expression, thus triggering LUAD cells at G2/M‐phase arrest. Consistent with this, we evaluated the expression levels of cell‐cycle biomarkers and found that bioinformatics analysis combined with N^6^‐methyladenosine (methylation at the N6 position in adenosine) RNA immunoprecipitation‐qPCR assay indicated that *GPR133* expression was down‐regulated by this modification. Moreover, we observed that methyltransferase‐like 3 was impaired in LUAD, and that it is able to significantly increase levels of *GPR133* by enhancing its RNA stability. In conclusion, we found that *GPR133* expression was down‐regulated in LUAD via N^6^‐methyladenosine modification. Increasing *GPR133* levels could suppress LUAD cell proliferation and tumor growth.

## Graphical abstract

G protein‐coupled receptor 133 (GPR133) is expressed at low levels in lung adenocarcinoma (LUAD). Furthermore, GPR133 inhibits LUAD cell proliferation and tumor growth. Mechanistically, GPR133 induces G2/M‐phase arrest by inhibiting cyclin B1 and enhancing p21 expression. Moreover, we demonstrated that METTL3 could regulate GPR133 in LUAD cells. Together, these results imply that GPR133 affects LUAD progression via regulation of cell proliferation.

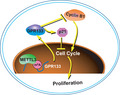

**Abbreviations**
GAPDH: glyceraldehyde‐3 phosphate dehydrogenase
GEO: Gene Expression Omnibus
GEPIA: Gene Expression Profiling Interactive Analysis
GO: Gene Ontology
GPCR: G protein‐coupled receptor
GPR133: G protein‐coupled receptor 133
GSEA: gene set enrichment analysis
KEGG: Kyoto Encyclopedia of Genes and Genomes
LUAD: lung adenocarcinoma
m^6^A: N^6^‐methyladenosine
METTL3: methyltransferase‐like 3
OS: overall survival
qRT‐OCR: quantitative RT‐PCR
SD: standard deviation
TCGA: The Cancer Genome Atlas

Lung adenocarcinoma (LUAD), which develops along the outer edges of the lungs within glandular cells in the small airways, accounts for approximately 40% of all lung cancer cases and constitutes a major cause of cancer mortality worldwide [1]. Recent efforts have focused on identifying biomarkers for the diagnosis and treatment of LUAD. Several genes, such as epidermal growth factor receptor (*EGFR*), anaplastic lymphoma kinase (*ALK*) and reactive oxygen species (*ROS*), were found to exhibit genomic mutations in LUAD [2], while other research found that molecularly targeted therapies directed at receptor tyrosine kinases were clinically successful. Studies are also currently being conducted on other targeted therapies directed against alterations in the *KRAS*, *ERBB2*, *BRAF*, *MET*, *RET*, *NTRK1* and *NTRK2* genes [3]. Encouragingly, immune checkpoint inhibitors targeting programmed cell death 1 receptor/programmed death‐ligand 1 mediated immunosuppression, showing efficacy in up to 30% of patients with LUAD [4]. However, many patients with LUAD have no common genetic mutation or acquire resistance to epidermal growth factor receptor tyrosine kinase inhibitors, even experiencing no response to immune checkpoint inhibition therapy. Thus, it is urgent to advance the understanding of the regulatory mechanisms involved in the development and progression of LUAD. Also, more sensitive novel biomarkers need to be identified for early diagnosis and therapeutic purposes.

The adhesion family forms a large branch of the pharmacologically important superfamily of G protein‐coupled receptors (GPCRs), which play important roles in receptor recognition, signal transduction, the cell cycle and cell differentiation [5]. G protein‐coupled receptor 133 (*GPR133*), also known as *ADGRD1*, is an orphan adhesion GPCR member. GPR133 contains a large N‐terminal extracellular domain, with a signal peptide and a pentraxin/concanavalin A domain [6]. Research suggests that the height and length of the R–R interval in the adult cardiac electrical cycle are related to the single‐nucleotide polymorphisms of *GPR133* [7, 8]. Notably, GPR133 correlates with altered bone mineral density in mouse knockouts, suggesting that it is a causal genetic driver of such disease in humans [9]. GPR133 expression increased as a function of World Health Organization grade and peaks in glioblastoma [10]. However, the role of GPR133 in LUAD remains unknown.

Here, we conducted a bioinformatics analysis of down‐regulated genes in LUAD using several databases, focusing in particular on GPR133. Our investigation demonstrated that GPR133 expression was decreased in LUAD and positively related to better outcomes among patients with LUAD. Moreover, increased GPR133 expression may significantly suppress the proliferation of LUAD cells *in vitro* and *in vivo*.

## Materials and methods

### Bioinformatics analysis

The mRNA expression profiles and outcomes of patients with LUAD were obtained from The Cancer Genome Atlas (TCGA) data portal (https://tcga‐data.nci.nih.gov/tcga/) and Gene Expression Omnibus (GEO) datasets (https://www.ncbi.nlm.nih.gov/). The down‐regulated genes with fold changes of two or more and *P* < 0.05 were chosen during subsequent analysis. We merged the earlier down‐regulated genes for intersection and focused on the *GPR133* gene. Meanwhile, the r software (R Foundation for Statistical Computing, Vienna, Austria) was used to match *GPR133* expression levels with the outcomes of patients with LUAD via TCGA data. Similarly, we analyzed *GPR133* expression levels and the prognosis of patients with LUAD using GEO datasets. Moreover, gene set enrichment analysis (GSEA) was implemented via GSEA version 2.2.2 (http://www.broadinstitute.org/gsea) to investigate the biological characteristics of *GPR133*. The genes correlated with *GPR133* were downloaded from the cBioPortal for Cancer Genomics (https://www.cbioportal.org/) and placed into the DAVID Bioinformatics Resources database (https://david.ncifcrf.gov/) to predict their function by Gene Ontology (GO) and Kyoto Encyclopedia of Genes and Genomes (KEGG) analysis. The N^6^‐methyladenosine (m^6^A) sites on the *GPR133* transcript were analyzed using the SRAMP online software program (http://www.cuilab.cn/sramp). The expression pattern of methyltransferase‐like 3 (*METTL3*) and the relationship between *METTL3* and *GPR133* were estimated using Gene Expression Profiling Interactive Analysis (GEPIA) data (http://gepia.cancer‐pku.cn/).

### Cells and clinical samples

The BEAS‐2B human bronchial epithelial cell line and several kinds of LUAD cells (A549, H1299, H1650, H1975 and PC9) were cultured in Roswell Park Memorial Institute (RPMI) 1640 (Gibco Laboratories, Gaithersburg, MD, USA) supplemented with 10% fetal bovine serum (Gibco Laboratories), incubated at 37 °C with 5% CO_2_ in a humidified incubator. A total of 28 cases of LUAD tissue samples and matched adjacent normal tissues were collected from patients in the People’s Hospital of Wuzhou between December 2011 and March 2019. There were 14 male and 14 female patients. The average age is 53.78 ± 4.67 years. Another cohort of normal tissues (*n* = 16, 5 female and 11 male patients; average age is 39.41 ± 10.27 years) and LUAD tissues (*n* = 63, 25 female and 38 male patients; the average age is 64.39 ± 9.17 years) with prognosis information was compiled from the biological resource specimen bank of the People’s Hospital of Wuzhou. The written informed consent was obtained from each patient for use of their tissue samples in research. This study was approved by the ethics committee of the People’s Hospital of Wuzhou.

### Transfection, quantitative RT‐PCR and western blot assays

For transfection assay, GPR133‐restored expression plasmid, METTL3 overexpression plasmid and vector plasmid were obtained from GeneCopoeia Biotechnology (Rockville, MD, USA). Lipofectamine 3000 Reagent was obtained from Thermo Fisher Scientific (Waltham, MA, USA). Cells were seeded into a six‐well plate overnight. Per well in a six‐well plate, 3 μg plasmid was incubated with 5 μL P3000 in 250 μL Opti‐MEM. A total of 5 μL Lipofectamine 3000 was mixed with 250 μL Opti‐MEM. Then these two mixtures were mixed together and incubated for 20 min at room temperature. Finally, the mixtures were added into one plate with 1.5 mL complete culture. Forty‐eight hours later, the cells were harvested for other assays.

For quantitative RT‐PCR (qRT‐PCR) assay, cells were treated with TRIzol reagent (Invitrogen, Carlsbad, CA, USA), and total RNAs were extracted and reversely transcribed into cDNA. qRT‐PCR assays were performed to measure the expression levels of target genes. All primers used are listed in Table 1.

**Table 1:** Primers in this study. F, forward; R, reverse.

Primer name	Sequence (5′–3′)
GPR133‐F	AAAGTCCCGGAGTGATACTGA
GPR133‐R	TTGGTGAGATTCAAGGCTGTC
GAPDH‐F	ACAACTTTGGTATCGTGGAAGG
GAPDH‐R	GCCATCACGCCACAGTTTC
Cyclin D1‐F	GCTGCGAAGTGGAAACCATC
Cyclin D1‐R	CCTCCTTCTGCACACATTTGAA
Cyclin B1‐F	AATAAGGCGAAGATCAACATGGC
Cyclin B1‐R	TTTGTTACCAATGTCCCCAAGAG
CDK4‐F	ATGGCTACCTCTCGATATGAGC
CDK4‐R	CATTGGGGACTCTCACACTCT
p21‐F	TGTCCGTCAGAACCCATGC
p21‐R	AAAGTCGAAGTTCCATCGCTC
Cdc2‐F	GGATGTGCTTATGCAGGATTCC
Cdc2‐R	CATGTACTGACCAGGAGGGATAG
IGF2BP3‐F	ACGAAATATCCCGCCTCATTTAC
IGF2BP3‐R	GCAGTTTCCGAGTCAGTGTTCA

For western blot assay, total protein was obtained from cells with lysis buffer and protease inhibitor. Bicinchoninic acid methods (Thermo Fisher Scientific) were used to determine protein concentrations. Proteins were loaded in gel channels to begin SDS/PAGE; transferred into polyvinylidene fluoride or polyvinylidene difluoride membranes; and incubated with 5% BSA, primary antibodies and horseradish peroxidase as a secondary antibody, respectively. All primary antibodies were listed in the manner of name, catalog name, dilution rate: glyceraldehyde‐3 phosphate dehydrogenase (GAPDH; 5174, 1 : 1000; CST), GPR133 (DF4947, 1 : 500; Affinity), p21 (2947, 1 : 500; CST), cyclin B1 (12231, 1 : 500; CST), cyclin D1 (55506, 1 : 500; CST), CDK4 (12790, 1 : 600; CST), Cdc2 (9116, 1 : 500; CST) and METTL3 (86132, 1 : 600; CST). Finally, an ECL chemiluminescence kit (Millipore, Burlington, MA, USA) was used to evaluate protein levels in the samples.

### m^6^A RNA immunoprecipitation‐qPCR assay

The m^6^A sites of *GPR133* were validated using the EZ‐Magna RNA immunoprecipitation kit (Millipore) according to the users’ instructions. Cells were seeded into a 10‐cm cell culture dish for overnight. In brief, cells were collected and lysed by RNA immunoprecipitation lysis buffer for 20 min, then centrifuged at 13 000 rpm for 10 min. The supernatant was incubated with m^6^A antibody and normal rabbit immunoglobulin G overnight, respectively. RNAs were extracted from magnetic beads, and we analyzed the level of *GPR133* in the earlier groups by qRT‐PCR assay.

### Immunohistochemistry

Tissues were deparaffinized in xylene, rehydrated with graded alcohol and boiled in 0.01 m citrate buffer (pH 6.0). Subsequently, the tissues were treated with 0.3% hydrogen peroxide, followed by normal goat serum.

All sections were incubated with the primary antibodies (GPR133, 1 : 100) overnight and followed by the biotinylated second antibody, streptavidin alkaline phosphatase, each for 10 min. Then the sections were then counterstained with hematoxylin, dehydrated and mounted. All immunohistochemical staining was evaluated and scored by at least two independent pathologists. The scores were set into four groups: negative (0–3), weak positive (3–6), middle positive (6–9) and strong positive (9–12).

### Cell viability assay

Cells were seeded in 96‐well plates at the concentration of 1500 cells per well. MTS was added into wells at 0, 1, 2, 3, 4 and 5 days, respectively, for 3 h. The absolute absorbance value at 490 nm (*A*_490__nm_) was measured using the Varioskan LUX system (Thermo Fisher Scientific).

### Colony formation assay and soft agar colony formation assay

For colony formation experiments, the cells were seeded into six‐well plates at a density of 750 cells per well and cultured. After 14 days, cells were fixed with 4% formaldehyde for 15 min and stained with 0.1% crystal violet for 10 min. Finally, the colonies were photographed, and any colonies larger than 1 mm (>50 cells per clone) were counted.

For soft agar colony formation assay, 1500 cells were fully mixed with complete RPMI 1640 medium and 0.75% agarose, then quickly placed on the complete RPMI 1640 medium curing layer with 1.5% agarose. Next, 0.5 mL of RPMI 1640 was added every 5 days to each well to feed cells. After 3 weeks, colonies larger than 50 μm were photographed.

### RNA stability assay

A549 cells with decreasing METTL3, H1299 cells with decreasing METTL3 concentrations and control group cells were seeded into six‐well plates, respectively. All cells were treated with 5 μg·mL^−1^ actinomycin D, and total RNA samples were obtained at 0, 1, 2 and 3 h. The mRNA expression level of *GPR133* was estimated by qRT‐PCR assay.

### Flow cytometry

A cell‐cycle staining kit was obtained from MultiSciences Biotech (Hangzhou, China), and cell cycles were analyzed by flow cytometry assay using this kit according to the manufacturer’s instructions. Cells were seeded in a six‐well plate, and starvation treatment was deployed overnight. Then cells were harvested, washed with cold phosphate‐buffered saline solution and incubated with DNA staining solution and permeabilization solution for 30 min at room temperature. Finally, cell samples were determined by using fluorescence‐activated cell sorting (Becton, Dickinson & Co., Franklin Lakes, NJ, USA).

### Animal experiments

All animal experiments were approved by the ethics committee of the People’s Hospital of Wuzhou. Four‐week‐old immunodeficient mice were purchased from the Guangdong Animal Center (Guangzhou, China). Animals were randomly divided into groups (*n* = 4). Each group had two male and two female patients. A total of 5 × 10^5^ cells were subcutaneously injected into the nude mice (*n* = 4 per group). Tumor growth was analyzed by measuring the tumor length (*L*) and width (*W*) and calculating the volume (*V*) using the formula: *V* = *LW* ^2^/2. The tumor tissues were then embedded in paraffin and analyzed.

### Statistical analysis

For all statistical tests, a two‐tailed *P* value <0.05 was considered to be statistically significant. Student’s *t*‐test and chi‐square test were performed to compare a single gene’s expression levels between two groups. Overall survival (OS) curves were estimated by Kaplan–Meier analysis.

## Results

### GPR133 was down‐regulated and associated with better prognosis in LUAD

**Fig. 1:**
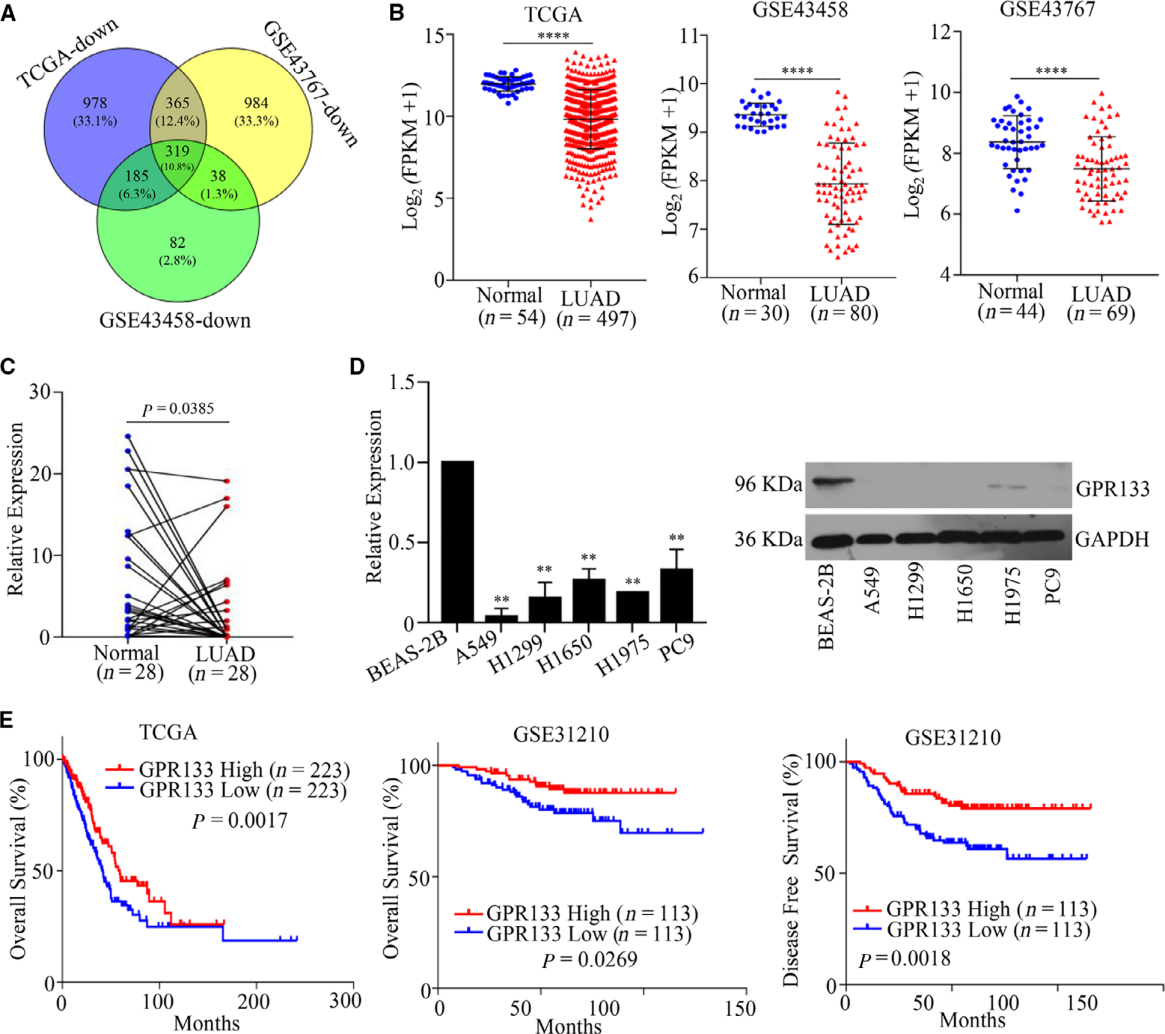
The expression pattern of *GPR133* in LUAD. (A) Gene expression information was downloaded from TCGA and GEO databases, and a Venn diagram was used to identify differentially expressed genes. (B) The expression pattern of *GPR133* in LUAD was displayed in TCGA and GEO databases vs. normal, ****P < 0.0001. (C) Total RNAs were obtained from 28 samples of LUAD tissue and matched adjacent normal tissues, and qRT‐PCR assays were implemented to detect *GPR133* expression in these tissues. Data were reported as mean ± standard deviation (SD) for three independent experiments, and statistical analysis was performed via Student’s *t*‐test. (D) The mRNA and protein expression levels of GPR133 in LUAD cells were measured by qRT‐PCR and western blot assays, respectively. Data were reported as mean ± SD for three independent experiments, and statistical analysis was performed via Student’s *t*‐test vs. BEAS‐2B, **P < 0.01. (E) Kaplan–Meier analysis was conducted according to *GPR133* expression in patients with LUAD from TCGA database and GSE31210 dataset.

To explore the tumorigenesis and development of LUAD, we searched for down‐regulated genes in LUAD among TCGA and GEO datasets and located a total of 319 genes down‐regulated in LUAD among TCGA, GSE43767 and GSE43458 datasets (Fig. 1A). Notably, the expression level of *GPR133* was much lower in 497 samples of LUAD tissue than that in 54 samples of normal tissue. *GPR133* expression was also decreased in 80 samples of LUAD tissue as compared with that in 30 samples of normal tissue in GSE43458; similar results were obtained in GSE43767 (Fig. 1B). Consistently, we found that *GPR133* expression was more significantly suppressed in 28 samples of LUAD tissue than in paired adjacent tissues (Fig. 1C). Further, we estimated GPR133 expression in LUAD cells, where not only the mRNA but also the protein expression level of GPR133 was much lower in LUAD cells than in normal BEAS‐2B cells (Fig. 1D). Next, we selected A549 and H1299 cells given their low expression of GPR133. To investigate the clinical implications of *GPR133* in LUAD, we conducted Kaplan–Meier analysis and found that patients with high *GPR133* expression levels had a better OS and disease‐free survival (Fig. 1E). In GSE31210, we performed the chi‐squared test and found that the expression of *GPR133* was related to the tumor stage of LUAD, but not with age, sex or smoking history (Table 2). Collectively, these results suggested that *GPR133* expression is decreased in LUAD and may be a potential biomarker of a better prognosis in patients with LUAD.

**Table 2:** The correlation between GPR133 expression and the clinical parameters of LUAD in GSE31210.

	Cases (*n*)	High		Low		*P* value
		Case (*n*)	Rate (%)	Case (*n*)	Rate (%)	
Age						
≥60	140	49	35.00	91	65.00	0.297
<60	106	44	41.51	62	58.49	
Sex						
Male	116	39	33.62	77	66.38	0.201
Female	130	54	41.54	76	58.46	
Smoking						
Yes	123	43	34.96	80	65.04	0.357
No	123	50	40.65	73	59.35	
Tumor stage						
I	169	68	40.24	101	59.76	0.000
II	58	6	10.34	52	89.66	

### Restored expression of GPR133 inhibited LUAD proliferation

**Fig. 2:**
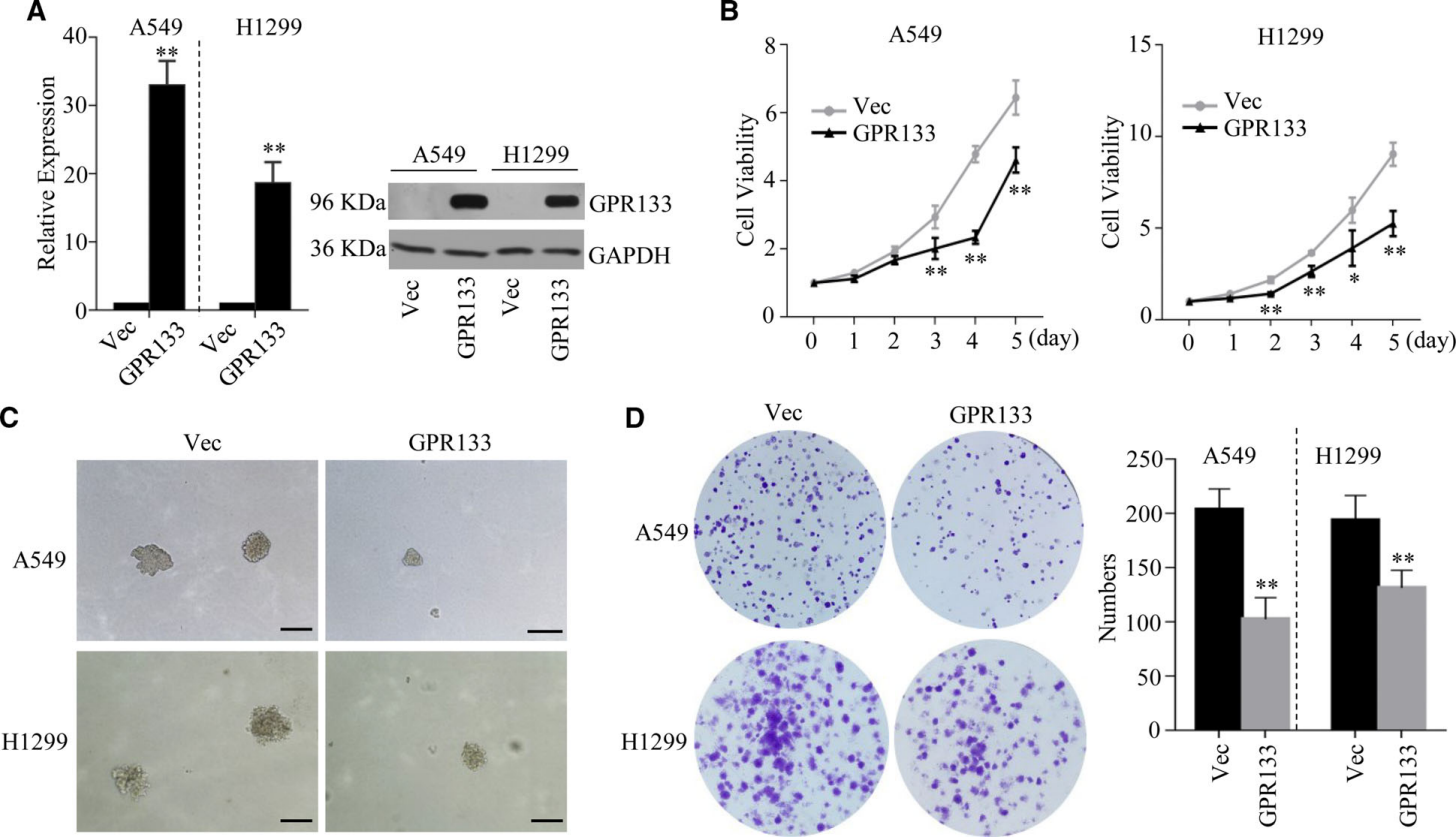
GPR133 suppressed cell proliferation in LUAD. *GPR133* overexpression plasmid was transfected into A549 and H1299 cells, respectively. (A) qRT‐PCR assays and western blot assays were performed to verify GPR133 in the mentioned cells. Data were reported as mean ± SD for three independent experiments, and statistical analysis was performed via Student’s *t*‐test vs. Vec, **P < 0.01. (B) The above cells were seeded into 96 wells, and MTS was added into each well at 0, 1, 2, 3, 4 and 5 days. The cell viability of each well was measured at *A*_490 nm_. Data were reported as mean ± SD for three independent experiments, and statistical analysis was performed via Student’s *t*‐test vs. Vec, *P < 0.05, **P < 0.01. Soft agar colony formation assay (C) and colony formation assay (D) were performed using the above cells. Data were reported as mean ± SD for three independent experiments, and statistical analysis was performed via Student’s *t*‐test vs. Vec, **P < 0.01. Scale bars: 100 μm. Vec, Vector.

To discover the effects of GPR133 on tumor progression, we first enhanced GPR133 expression in A549 and H1299 cells (Fig. 2A). Interestingly, the cell viability of both A549 and H1299 cells was sharply decreased by accelerating GPR133 in these cells (Fig. 2B). Consistent with this observation, colony formation and soft agar colony formation assays also demonstrated that LUAD cells with restored GPR133 expression formed significantly smaller and fewer colonies than the control group (Fig. 2C,D). These data indicate that GPR133 inhibited the proliferation ability of LUAD cells.

### GPR133‐triggered cell‐cycle arrest in LUAD cells

**Fig. 3:**
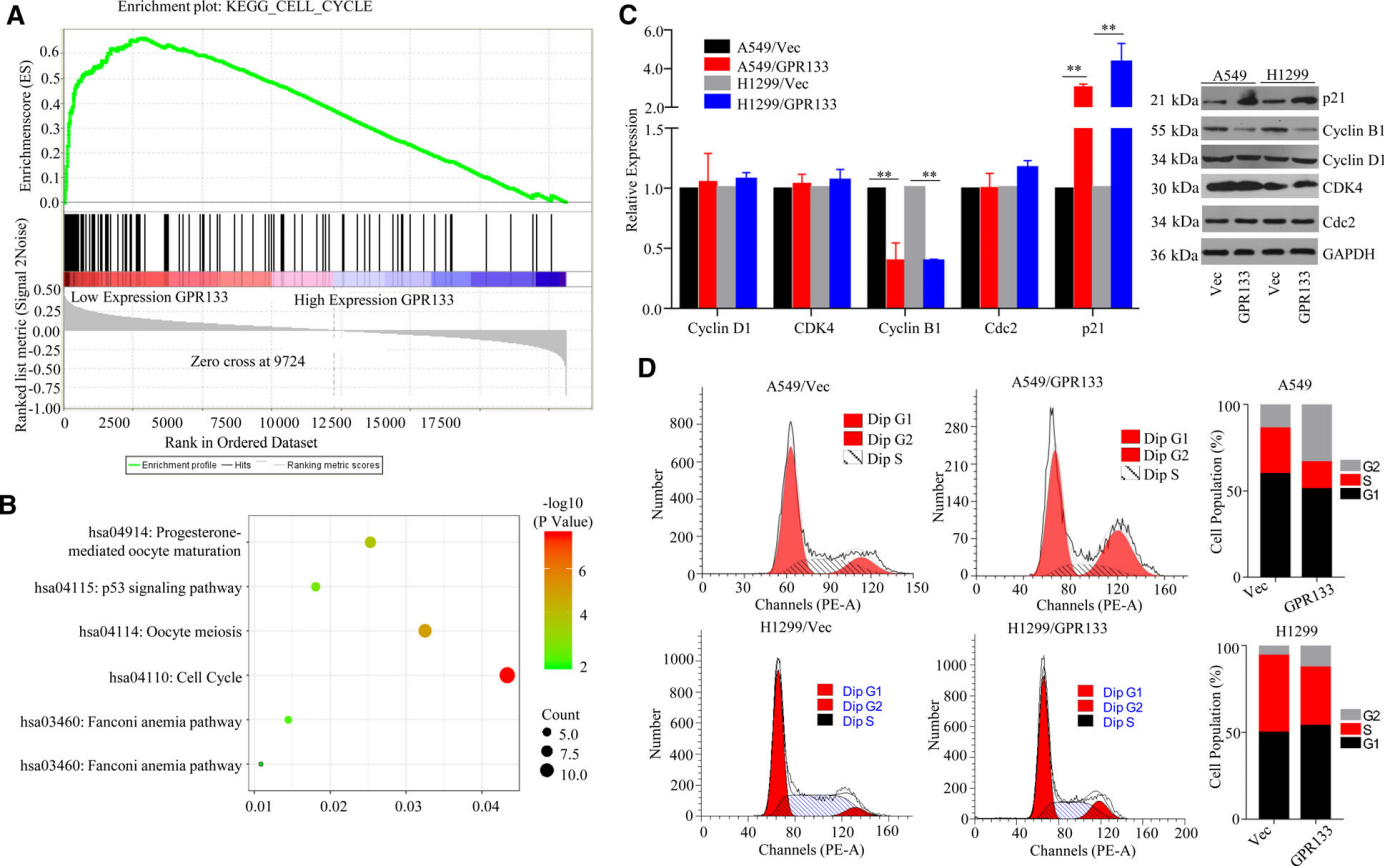
Effect of GPR133 on cell‐cycle distribution in LUAD cells. (A) GSEA assays were performed to explore the mechanism of GPR133 function in LUAD cells. (B) GPR133‐coexpressed genes were analyzed by GO and KEGG using the clusterprofiler software package on the r platform. (C) In LUAD cells with accelerating GPR133 and in control groups, the expression levels of cell‐cycle biomarkers were evaluated by qRT‐PCR and western blot assays. Data were reported as mean ± SD for three independent experiments, and statistical analysis was performed via Student’s *t*‐test vs. Vec, **P < 0.01. (D) Flow cytometry assays were conducted to detect the cell cycle in these cells.

GSEA analysis was conducted to discover the possible mechanism through which *GPR133* is involved in the proliferation of LUAD. The results revealed that *GPR133* expression was correlated with the cell cycle in LUAD (Fig. 3A). As shown in Fig. 3B, GO annotations suggested that *GPR133* coexpressed genes that were mainly involved in the cell cycle. Thus, we estimated the mRNA and protein expression levels of several biomarkers for the cell cycle. It was noted that GPR133 expression reduced cyclin B1 and enhanced p21 but had no effect on cyclin D1, CDK4 or Cdc2 (Fig. 3C). In addition, a flow cytometry assay was performed to measure the effect of GPR133 expression on the cell cycle of LUAD cells and revealed that overexpressing GPR133 triggered G2/M‐phase arrest in LUAD cells (Fig. 3D). Collectively, these results suggest that GPR133 plays a role in G2/M‐phase arrest in LUAD cells.

### GPR133 expression is enhanced by METTL3‐mediated m^6^A modification in LUAD

**Fig. 4:**
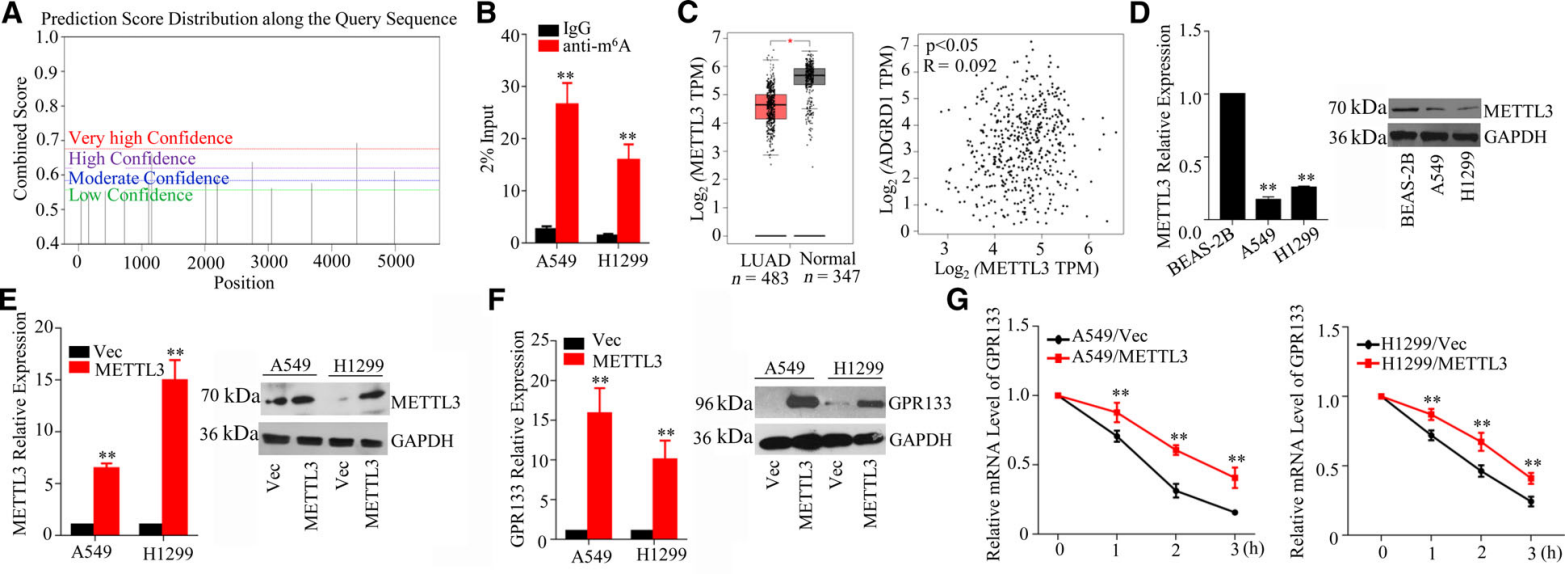
Identification of METTL3 targeting GPR133 in LUAD cells. (A) SRAMP presumed that the m^6^A was abundant in *GPR133* transcripts. (B) The cells were isolated and RNA immunoprecipitation assay was performed. m^6^A antibody was used to trigger target RNAs, while immunoglobulin G was used as a negative control. The expression of *GPR133* was determined in this experiment by qRT‐PCR assay. Data were reported as mean ± SD for three independent experiments, and statistical analysis was performed via Student’s *t*‐test vs. immunoglobulin G, **P < 0.01. (C) The expression pattern of *METTL3* in LUAD was exhibited. The relationship between *METTL3* and *GPR133* was analyzed by Pearson’s correlation coefficient using the GEPIA databank vs. normal, *P < 0.05. (D) The expression levels of METTL3 in BEAS‐2B, A549 and H1299 cells were detected by qRT‐PCR and western blot assays. Data were reported as mean ± SD for three independent experiments, and statistical analysis was performed via Student’s *t*‐test. BEAS‐2B, **P < 0.01. (E) *METTL3* overexpression plasmid was transfected into LUAD cells, and the mRNA and protein expression profiles of METTL3 were assessed by qRT‐PCR and western blot assays. Data were reported as mean ± SD for three independent experiments, and statistical analysis was performed via Student’s *t*‐test vs. Vec, **P < 0.01. (F) In LUAD cells with increasing METTL3 expression, the mRNA and protein expression levels of GPR133 were evaluated by qRT‐PCR and western blot assays. Data were reported as mean ± SD for three independent experiments, and statistical analysis was performed via Student’s *t*‐test vs. Vec, **P < 0.01. (G) The decay rate of *GPR133*’s mRNA at the indicated times after actinomycin D (5 µg·mL^−1^) treatment in A549 and H1299 cells with restored METTL3 expression was measured. Data were reported as mean ± SD for three independent experiments, and statistical analysis was performed via Student’s *t*‐test vs. Vec, **P < 0.01.

To discover the mechanism of GPR133 down‐regulation in LUAD, we analyzed the m^6^A sites on the *GPR133* transcript using SRAMP and predicted that several m^6^A sites exist on the *GPR133* transcript (Fig. 4A). We next performed m^6^A RNA immunoprecipitation‐qPCR assays to test this hypothesis, and the results revealed a substantial increase in the m^6^A level in A549 and H1299 cells (Fig. 4B). Moreover, we examined the correlation between the expression levels of *GPR133* and m^6^A writers in TCGA. As shown in Fig. 4C, *METTL3* expression was impaired in 483 samples of LUAD tissue and 347 samples of normal tissue (*P *< 0.01). Although TCGA and Genotype‐Tissue Expression (GTEx) datasets of LUAD showed weak positive correlation of METTL3 and GPR133 (*R* = 0.092), further cell line results strengthened it. Notably, qRT‐PCR and western blot assays revealed that the expression levels of METTL3 in A549 and H1299 cells were much lower than those in BEAS‐2B cells (Fig. 4D). On the basis of METTL3 expression, we transfected METTL3 overexpression plasmid into LUAD cells to restore an appropriate level of METTL3 expression (Fig. 4E). Actually, accelerating METTL3 may enhance not only the mRNA but also the protein expression levels of GPR133 (Fig. 4F). To verify whether METTL3 promoted the stability of *GPR133* mRNA, we used actinomycin D to observe the mRNA level of *GPR133*. Upon increasing the actinomycin D treatment time, the decay of *GPR133* mRNA was reduced in A549 and H1299 cells with overexpressed METTL3 as compared with in the respective control groups (Fig. 4G). Given these results, we presumed that METTL3 modulates *GPR133* in an m^6^A‐dependent manner.

### GPR133 inhibited LUAD tumor growth *in vivo*

**Fig. 5:**
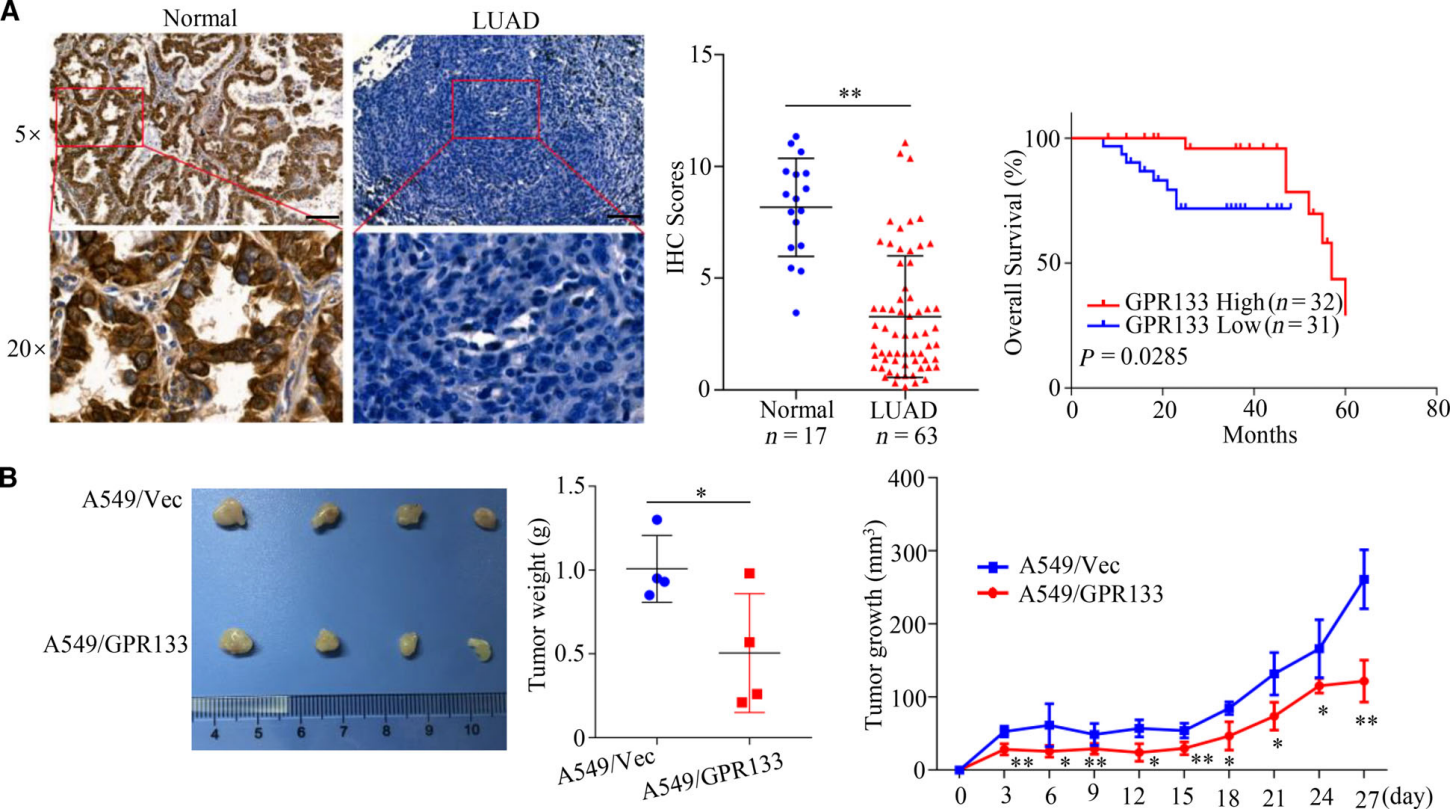
GPR133 inhibited tumor growth *in vivo*. (A) Immunohistochemistry assays were implemented to determine GPR133 expression in normal tissues and LUAD tissues. Data were reported as mean ± SD for three independent experiments, and statistical analysis was performed via Student’s *t*‐test vs. normal, **P < 0.01. The relationship between GPR133 expression and the prognosis of patients with LUAD was established by Kaplan–Meier analysis. Scale bars: 100 μm. (B) A total of 5 × 10^5^ A549 cells with overexpressed GPR133 and the control group were subcutaneously injected into nude mice (*n* = 4 per group), respectively. The tumor size was detected every 2 days; tumor weights were also measured, and the tumor growth curve was drawn. Data were reported as mean ± SD, and statistical analysis was performed via Student’s *t*‐test vs. Vec, *P < 0.05, **P < 0.01.

In brief, we estimated GPR133 expression patterns in LUAD by immunohistochemistry assay. The results revealed that GPR133 expression was down‐regulated in 63 samples of LUAD tissue and in 17 samples of normal tissue (Fig. 5A). Meanwhile, Kaplan–Meier analysis demonstrated that patients with LUAD with high GPR133 expression levels had a better outcome (Fig. 5A). To further investigate the effects of GPR133 on tumorigenicity *in vivo*, we subcutaneously injected A549 cells with increasing GPR133 expression into nude mice and assessed the subsequent tumor growth; ultimately, the results showed that overexpression of GPR133 could markedly suppress tumor growth *in vivo* (Fig. 5B). As compared with in the control group, restored GPR133 expression may significantly suppress both tumor weight and tumor growth (Fig. 5B). All these data support that GPR133 is a tumor inhibitor in LUAD.

## Discussion

*GPR133*, a member of the GPCR superfamily, regulates cell adhesion and cell metabolism. In this study, we provided the first evidence that *GPR133* expression is up‐regulated in LUAD by METTL3 in an m^6^A‐dependent manner. However, METTL3 was impaired in LUAD. Bioinformatics analysis combined with experiments suggested that GPR133 expression is positively related to a better prognosis among patients with LUAD. Functionally, GPR133 suppressed the proliferation of LUAD cells both *in vivo* and *in vitro*. Importantly, we showed that GPR133 triggered G2/M‐phase arrest in LUAD cells. Hence targeting GPR133 might represent a novel strategy to treat LUAD.

Recently, m^6^A has been discovered to be a reversible RNA methylation factor. This dynamic RNA methylation factor is enriched around stop codons, in 3′ untranslated regions and within internal long exons [11]. The act of m^6^A modification affects fundamental aspects of mRNA metabolism, resulting in posttranscriptional dysregulation of gene expression relating to cell differentiation, cell homeostasis, the cellular response to stress and cancer [12]. In this modification system, m^6^A ‘writers’ are composed of core catalytic components (METTL3/methyltransferase‐like 14) to install m^6^A modification [13]. Alkylation repair homolog protein 5 (ALKBH5) and fat mass and obesity‐associated protein, which are termed as m^6^A erasers, focus on removing m^6^A modification [14, 15]. The function of m^6^A is executed by m^6^A ‘readers’ that bind to m^6^A directly (YT521‐B homology domain‐containing proteins, Eukaryotic initiation factor 3 and Insulin‐like growth factor 2 mRNA‐binding proteins) or indirectly (HNRNPA2B1) [16, 17, 18].

METTL3 has been recognized as an essential factor in conditions including diabetes, cancers and cardiovascular disease [19, 20]. The METTL3‐mediated m^6^A modification on AFF4 could promote its expression. In addition, AFF4 is bound to the promoter of MYC. As such, the METTL3/AFF4/MYC axis contributes to bladder cancer tumorigenesis [21]. METTL3 targeted the 3′ untranslated region of *HK2* mRNA. Moreover, METTL3 recruited YTHDF1 to enhance *HK2* stability, thereby promoting the Warburg effect of cervical cancer [22]. Consistent with the aforementioned study, we found that METTL3 was expressed at low levels in LUAD. *METTL3* was positively correlated with *GPR133* via the GEPIA databank. Moreover, the overexpression of METTL3 may significantly enhance the mRNA stability of *GPR133*.

To discover the function of GPR133 expression in LUAD, we performed cell viability and colony formation assays, where the results showed that increasing the GPR133 expression sharply suppressed the proliferation of LUAD cells. Further investigation determined that GPR133 inhibited tumor growth in animal experiments of LUAD. However, it was reported that GPR133 was selectively expressed in hypoxic regions of GBM, while GPR133 knockdown abrogated tumor initiation [10]. Several genes, including GPR133, were found to be up‐regulated in gastrointestinal stromal tumors, but their functions remain unknown [23].

To discover the potential regulatory mechanism of GPR133 in LUAD, we performed GO and KEGG analyses based on *GPR133*‐related genes. The results indicated that the cell cycle was the most likely possible regulatory mechanism of GPR133 in LUAD. The cell cycle is a key event of cells, and targeting the cell cycle may be an important approach in cancer therapy [24]. It is well known that cell‐cycle machinery is controlled by cyclin‐dependent kinase (CDK), cyclins, and CDK‐inhibitory proteins [25]. Hence we quantified the mRNA and protein expression levels of cell‐cycle biomarkers in LUAD cells with increasing GPR133 expression. Our results indicated that GPR133 inhibited cyclin B1 and enhanced p21 expression in LUAD cells. Notably, cyclin B1 and p21 are famous G2/M‐phase biomarkers. Next, we performed a flow cytometry assay to discern that *GPR133* significantly induced G2/M‐phase arrest in LUAD cells. Consistently, PP9 (a natural steroidal saponin) was reported to effectively induce G2/M‐phase arrest by up‐regulating p21 and suppressing cdc25C, cyclin B1 and cdc2 [26]. Evidently, avasimibe dose‐dependently inhibited the proliferation of U251 and U87 human glioblastoma cells. Further research revealed that avasimibe suppressed the expression of CDK2, cyclin E1, CDK4, cyclin D, CDK1, cyclin B1, Aurora A and PLK1, while inducing the expression of p53, p21, p27 and GADD45A [27].

Our encouraging data presented herein lay the foundation for further research of GPR133 in LUAD as a therapeutic target. Indeed, we are currently conducting experiments geared toward further target validation, as well as toward developing GPR133 inhibitors via small biomolecules.

## Conflict of interest

The authors declare no conflict of interest.

## Author contributions

GW and DZ performed most of the experiments and analyzed results. JX and ZL did the bioinformatics analysis. XL and SZ analyzed the data. ZZ designed the research. GW wrote the paper. ZZ and XL revised the paper.

## Data Availability

All data are included in the manuscript.
